# P-112. Sotrovimab for Pre-exposure Prophylaxis against SARS-CoV2 in a Vulnerable Patient Population: Results from the PROTECT-V trial

**DOI:** 10.1093/ofid/ofaf695.340

**Published:** 2026-01-11

**Authors:** Michael Chen-Xu, Wendi Qian, Kimia Kamelian, Giorgio Trivioli, Davinder Dosanjh, Francis Dowling, Rakshya Adhikari, Jennifer Han, Thomas F Hiemstra, Alex G Richter, Ravindra K Gupta, Rona M Smith

**Affiliations:** University of Cambridge, Cambridge, England, United Kingdom; Cambridge University Hospitals NHS Foundation Trust, Cambridge, England, United Kingdom; University of Cambridge, Cambridge, England, United Kingdom; University of Cambridge, Cambridge, England, United Kingdom; University of Birmingham, Birmingham, Northern Ireland, United Kingdom; Cambridge University Hospitals NHS Foundation Trust, Cambridge, England, United Kingdom; Cambridge University Hospitals NHS Foundation Trust, Cambridge, England, United Kingdom; GlaxoSmithKline, Collegeville, Pennsylvania; Cambridge University Hospitals NHS Foundation Trust, Cambridge, England, United Kingdom; University of Birmingham, Birmingham, Northern Ireland, United Kingdom; University of Cambridge, Cambridge, England, United Kingdom; University of Cambridge, Cambridge, England, United Kingdom

## Abstract

**Background:**

Despite introduction of vaccination against SARS CoV-2, there remains a need for pre-exposure prophylaxis in patients that mount suboptimal vaccine responses. Sotrovimab is a recombinant human monoclonal antibody directed against the spike protein of SARS-CoV-2. The COMET-ICE study demonstrated that 500mg intravenous (IV) sotrovimab significantly reduced all-cause hospitalization or death in high-risk non-hospitalised patients with mild/moderate COVID-19 infection.

**Figure:**
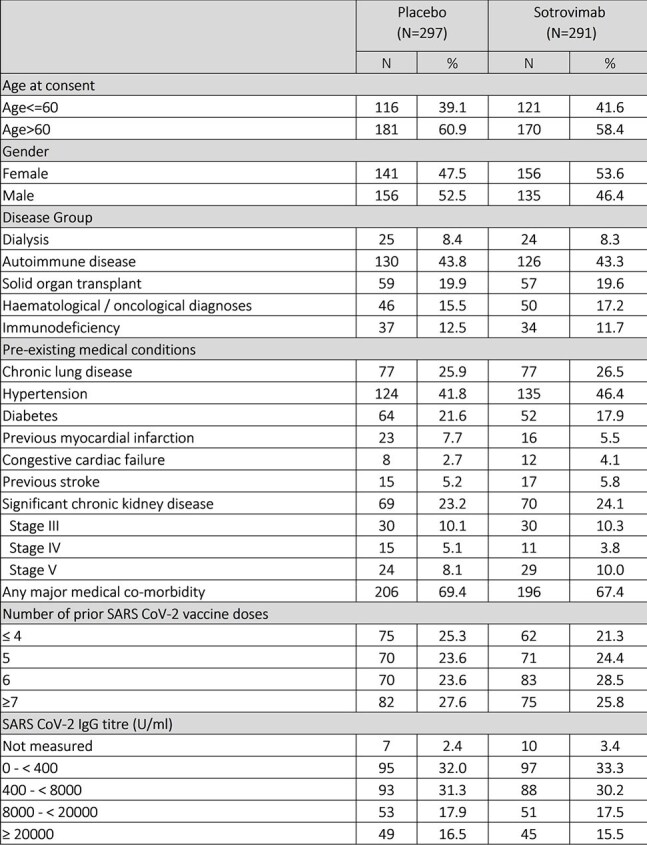

**Table 2:**
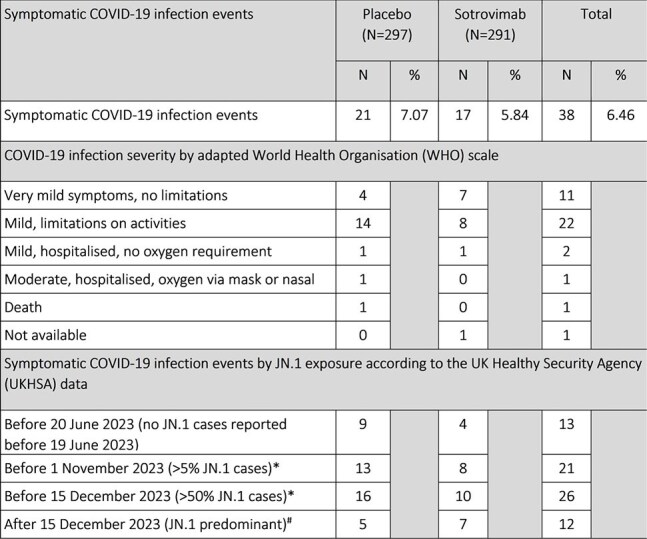
Number and severity of COVID-19 infections at week 12 (primary efficacy endpoint) *cumulative cases of COVID-19 infection, so includes cases reported in row(s) above. # cases reported before and after 15 December 2023 equate to the total number of symptomatic COVID-19 infections at week 12.

**Methods:**

PROTECT-V is a platform trial evaluting prophylactic interventions against SARS-CoV2 infection in vulnerable adult patients at high risk of COVID-19 infection and its complications, including dialysis patients, transplant recipients, those with autoimmune diseases, underlying immunodeficiency or haematological/oncological diagnoses.

Sotrovimab was the second agent added to the platform. Participants were randomized 1:1 to one dose of IV sotrovimab 2000mg or matched placebo. The primary endpoint was confirmed symptomatic COVID-19 infection at week 12.

**Table 3:**
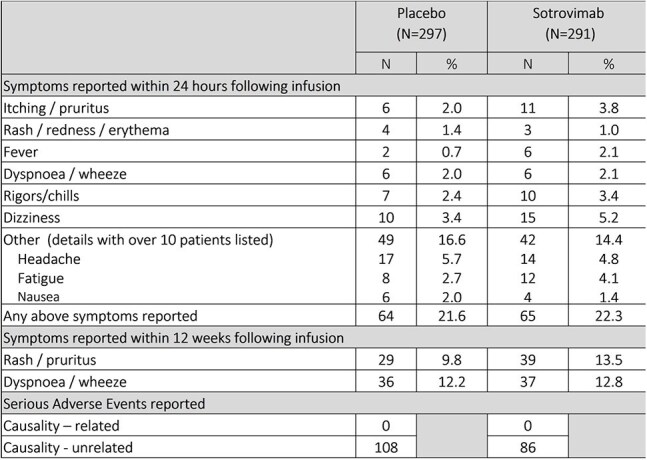
Safety and Tolerability of 2000mg intravenous Sotrovimab

**Results:**

619 (314 placebo, 305 sotrovimab) patients in the UK were randomized between August 2022 and May 2024. 588 (297 placebo, 291 sotrovimab) received investigational medicinal product infusion. Overall, median age was 64.4 years and 50.5% were female (see Table 1: Baseline characteristics).

At week 12, 21 symptomatic COVID-19 infections were observed in the placebo group and 17 in the sotrovimab group with a risk ratio of 0.80 (95% CI 0.42– 1.53) adjusting for age and disease group. 4/38 (10.5%) patients were hospitalised (3 placebo, 1 sotrovimab). In view of the 250-fold reduction in EC50 with sotrovimab on pseudovirus testing for JN.1, a pre-specified efficacy analysis according to exposure period was performed (Table 2).

Headache, dizziness and skin symptoms were the most common adverse events reported within 24 hours of infusion (Table 3).

**Conclusion:**

Although safe and well tolerated, 2000mg sotrovimab did not demonstrate benefit over placebo for the prevention of symptomatic SARS-CoV2 infection at 12 weeks. There was potential signal of benefit for sotrovimab before the emergence of the JN.1 variant in late 2023 that rapidly became the dominant circulating variant in the UK at that time.

**Disclosures:**

Michael Chen-Xu, MBChB, MRCP, MPH, GlaxoSmithKline: Grant/Research Support Davinder Dosanjh, n/a, Astrazeneca: Employee Jennifer Han, MD, GlaxoSmithKline: Employee Thomas F. Hiemstra, n/a, GlaxoSmithKline: Stocks/Bonds (Public Company)|Novartis: Employee|Novartis: Stocks/Bonds (Public Company) Alex G. Richter, n/a, CSL Behring: Honoraria Rona M. Smith, MD MRCP, AstraZeneca: Advisor/Consultant|AstraZeneca: Honoraria|GlaxoSmithKline: Grant/Research Support|Vifor Pharma: Honoraria

